# Peroxiredoxin 3 Is a Redox-Dependent Target of Thiostrepton in Malignant Mesothelioma Cells

**DOI:** 10.1371/journal.pone.0039404

**Published:** 2012-06-25

**Authors:** Kheng Newick, Brian Cunniff, Kelsey Preston, Paul Held, Jack Arbiser, Harvey Pass, Brooke Mossman, Arti Shukla, Nicholas Heintz

**Affiliations:** 1 Department of Pathology, University of Vermont College of Medicine, Burlington, Vermont, United States of America; 2 BioTek Instruments, Winooski, Vermont, United States of America; 3 Department of Dermatology, Emory University School of Medicine, Atlanta, Georgia, United States of America; 4 Department of Cardiothoracic Surgery, New York University Langone Medical Center, New York, New York, United States of America; 5 Vermont Cancer Center, University of Vermont, Burlington, Vermont, United States of America; Virginia Commonwealth University, United States of America

## Abstract

Thiostrepton (TS) is a thiazole antibiotic that inhibits expression of FOXM1, an oncogenic transcription factor required for cell cycle progression and resistance to oncogene-induced oxidative stress. The mechanism of action of TS is unclear and strategies that enhance TS activity will improve its therapeutic potential. Analysis of human tumor specimens showed FOXM1 is broadly expressed in malignant mesothelioma (MM), an intractable tumor associated with asbestos exposure. The mechanism of action of TS was investigated in a cell culture model of human MM. As for other tumor cell types, TS inhibited expression of FOXM1 in MM cells in a dose-dependent manner. Suppression of FOXM1 expression and coincidental activation of ERK1/2 by TS were abrogated by pre-incubation of cells with the antioxidant N-acetyl-L-cysteine (NAC), indicating its mechanism of action in MM cells is redox-dependent. Examination of the mitochondrial thioredoxin reductase 2 (TR2)-thioredoxin 2 (TRX2)-peroxiredoxin 3 (PRX3) antioxidant network revealed that TS modifies the electrophoretic mobility of PRX3. Incubation of recombinant human PRX3 with TS *in vitro* also resulted in PRX3 with altered electrophoretic mobility. The cellular and recombinant species of modified PRX3 were resistant to dithiothreitol and SDS and suppressed by NAC, indicating that TS covalently adducts cysteine residues in PRX3. Reduction of endogenous mitochondrial TRX2 levels by the cationic triphenylmethane gentian violet (GV) promoted modification of PRX3 by TS and significantly enhanced its cytotoxic activity. Our results indicate TS covalently adducts PRX3, thereby disabling a major mitochondrial antioxidant network that counters chronic mitochondrial oxidative stress. Redox-active compounds like GV that modify the TR2/TRX2 network may significantly enhance the efficacy of TS, thereby providing a combinatorial approach for exploiting redox-dependent perturbations in mitochondrial function as a therapeutic approach in mesothelioma.

## Introduction

Malignant mesothelioma (MM) is a type of cancer originating from the mesothelial lining of the pleural and peritoneal cavities [1]. It is a deadly malignancy primarily associated with exposure to asbestos, with an annual incidence of 2000–3000 cases in the United States [1]. Due to long latency periods, the risk of developing MM increases with age [1], and the incidence of MM is expected to rise in regions where asbestos use has been banned, as well as in countries where protection from occupational exposures is presently lacking [2], [3]. Pleural malignant mesothelioma is the most common type of mesothelioma [3], and it primarily affects men, with a men-to-women ratio of 5∶1. Effective therapy for MM is lacking, with average survival estimated at less than 2 years [1].

We are interested in developing new approaches to treating MM, and have begun investigation of FOX family proteins in this disease entity. The FOX (for forkhead box) family encompasses over 100 proteins that play important roles in development, cell proliferation, cell survival, metabolism, stress responses and aging (reviewed in [4], [5]). The forkhead superfamily of transcription factors is characterized by a common DNA binding domain first identified in the *Drosophila melanogaster* forkhead gene product [4], [5], [6]. Several members of the FOX family of transcriptional regulators, including FOXO3a and FOXM1, have emerged as important therapeutic targets in human malignancies [5].

The FOX family member FOXM1 regulates the expression of genes involved in cell survival and cell cycle progression, including S phase entry [4], [7] and transition through mitosis [8], [9], [10]. Alternative splicing results in three protein isoforms: FOXM1A, which acts as a transcriptional repressor [11], and FOXM1B and FOXM1C, which are transcriptional activators [4]. FOXM1 is not expressed in non-cycling cells and is induced in response to growth factor stimulation via the E2F pathway [12]. FOXM1 has an N-terminal auto-inhibitory domain, and N-terminal deletion mutants of FOXM1C are constitutively active, whereas activation of the full length protein requires growth factor signaling [4]. FOXM1 enters the nucleus during G2 in an ERK-dependent manner [13], and is degraded during exit from mitosis by APC/Cdh1, an event required for regulated entry into the next S-phase [9], [10]. Depletion of FOXM1 in mouse models of cancer markedly impedes tumor progression (reviewed in [4], [6]), indicating FOXM1 is an important factor in tumor progression. The oncogenic splice isoforms FOXM1B and/or FOXM1C are over-expressed in all carcinomas examined to date [4], but not in quiescent tissues, suggesting FOXM1 may represent a therapeutic target in many human solid tumor types.

Chronic oxidative stress has long been recognized as a phenotypic feature of many cancers [14], [15], [16], and certain tumors appear to rely on the enhanced production of reactive oxygen species (ROS) for cell proliferation. FOXM1 has emerged recently as an important cell cycle regulator that sits at the interface between oxidative stress, aging, and cancer [4], [6], [17]. Expression of FOXM1 is inhibited by antioxidants and induced by hydrogen peroxide (H_2_O_2_), albeit through unknown mechanisms [17]. FOXM1 counteracts oncogenic Ras-induced oxidative stress through the up-regulation of antioxidant enzymes that include mitochondrial manganese superoxide dismutase (MnSOD), catalase, and peroxiredoxin 3 (PRX3) [17]. A screen for small molecules that inhibit the transcriptional activity of FOXM1 identified siomycin A [18], a thiazole antibiotic very similar to thiostrepton (TS), one of a large family of multicyclic peptide antibiotics produced by diverse bacteria [18]. TS induces cell cycle arrest and selectively kills breast cancer cells through down-regulation of FOXM1 protein and RNA expression [19], and acts synergistically with other agents to promote tumor cell apoptosis [20]. TS has modest anti-tumor activity in a xenoplant model of breast cancer in mice [21].

While TS inhibits protein synthesis through binding to ribosomes in prokaryotes [22], its mechanism of action in mammalian cells is not well understood. TS has been reported to bind directly to FOXM1 [23] and to inhibit proteosome activity leading to induction of the cyclin-dependent kinase inhibitor p21 [24], [25], [26]. In melanoma cells, but not primary melanocytes, TS induces oxidative stress and impairs the activity of the proteosome, eliciting proteotoxic stress, upregulation of heat shock and stress response gene expression, and apoptosis [27]. Here we have investigated the mechanism of action of TS in malignant mesothelioma (MM) cells, and report that TS acts via redox-dependent mechanism that includes covalent adduction of PRX3, a mitochondrial peroxidase linked to suppression of apoptosis [28], [29]. In cancer cells elevated expression of the mitochondrial thioredoxin reductase 2 (TR2)-thioredoxin 2 (TRX2)-PRX3 antioxidant network is an adaptive response to increased mitochondrial oxidative stress. Since inhibiting expression of PRX3 sensitizes cells to apoptosis [28], [29], this mitochondrial antioxidant enzyme has emerged as a therapeutic target in cancer [29]. Here we show that disabling PRX3 by TS correlates with increased mitochondrial oxidant production, inhibition of FOXM1 expression, and cell death. The cationic triphenylmethane gentian violet (GV), which inhibits expression of TRX2 [30], the mitochondrial oxidoreductase required for reduction of PRX3 during its catalytic cycle, markedly enhances both modification of PRX3 and the cytotoxic activity of TS. These studies provide a combinatorial approach for inhibiting FOXM1 expression and impairing tumor cell viability that may prove particularly useful in MM and other tumor types characterized by aberrant mitochondrial oxidant production.

## Results

### MM Tumors and Cells Express FOXM1

**Figure 1:**
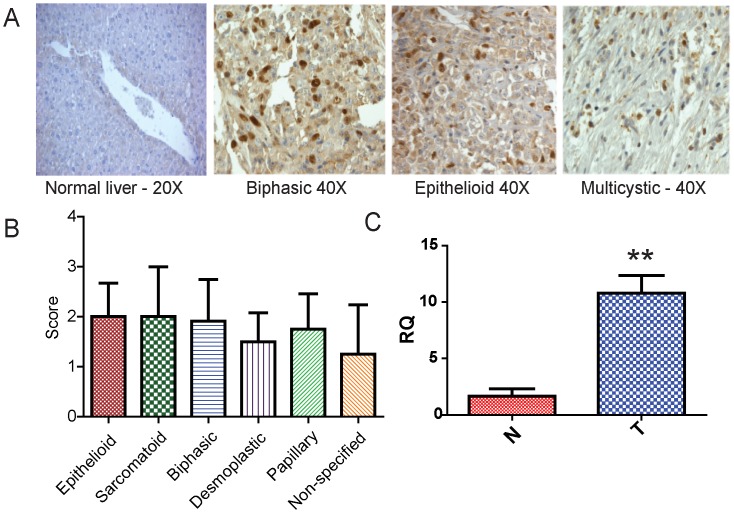
Human MMs express FOXM1. A) Paraffin-embedded sections from human MM tissue specimens were examined for FOXM1 expression using immunohistochemistry; shown are representative results for the indicated tumor types at a 1∶3000 dilution of primary antibody, which accentuates nuclear localization of FOXM1. No signal for FOXM1 was observed in normal human liver. B) Nuclear expression of FOXM1 was scored for the indicated tumor types, using 0 =  no positive nuclei, 1 = <5% positive nuclei, 2 = >5% and <50% positive nuclei, and 3 = >50% positive nuclei. Significant levels of nuclear FOXM1 expression were observed in all MM tumor types. There was no significant difference in nuclear FOXM1 expression between mesothelioma tumor types. C) Total RNA was extracted from normal mesothelial tissue (N) or mesothelioma (T) from four patients and examined for FOXM1 mRNA expression relative to HPRT using qRT-PCR. Using the Student’s t-test, relative expression (RQ) for FOXM1 transcript in tumor tissue was increased as compared to normal mesothelium (p  = 0.0017).

To determine if FOXM1 is a relevant therapeutic target in MM, we examined FOXM1 expression in tissue arrays of human MM tumor specimens with a polyclonal FOXM1 antibody at multiple dilutions (1∶1000, 1∶2000 and 1∶3000), as in another study [31]. At dilutions of 1∶3000, there was no staining in control tissues such as liver (Fig. 1A). Since the significance of cytoplasmic staining of FOXM1 is not well understood, specimens were scored for nuclear staining on a scale of 0–3. Specimens stained at the 1∶3000 dilution showed predominantly nuclear staining and were scored by two observers: 0 =  no positive nuclei, 1 = <5% positive nuclei, 2 = >5% and <50% positive nuclei, and 3 = >50% positive nuclei. Regardless of subtype, the majority of MM tumors expressed nuclear FOXM1 in 50% or more of the cells (Fig. 1B). In general, the epithelioid compartments of mesothelioma tumor specimens displayed nuclear or cytoplasmic staining, with little staining of connective tissue elements. Quantification of FOXM1 mRNA expression in MM tumor specimens and matched control tissues from four individuals showed that FOXM1 mRNA is elevated in human MM (Fig. 1C), confirming the results obtained by immunohistochemistry.

**Figure 2:**
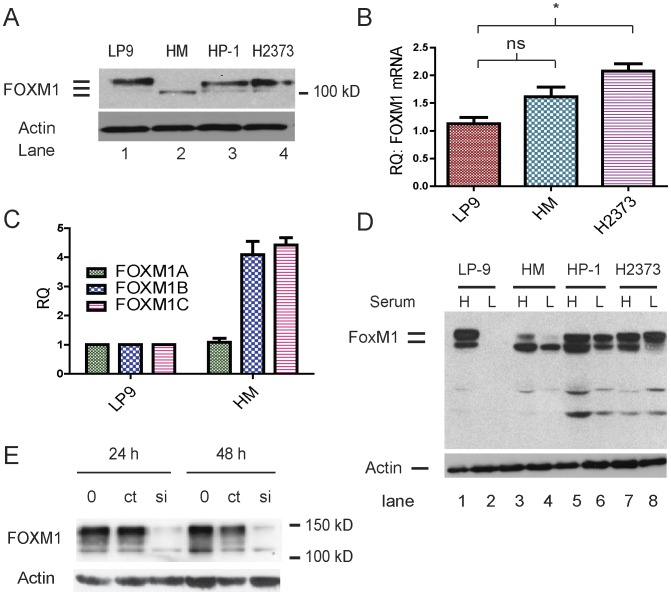
Expression of FOXM1 in LP9 mesothelial cells and MM cells lines. A) FOXM1 expression was examined by immunoblotting of cell extracts; note that LP9 expresses a larger isoform of FOXM1 than the MM cell lines. B) Total RNA was extracted from the indicated cell lines in log phase growth, and FOXM1 mRNA expression relative to HPRT (RQ) was determined by qRT-PCR. C) Isoform-specific RT-PCR was used to examine expression of FOXM1A, FOXM1B, and FOXM1C mRNA in the indicated cell lines. HM cells expressed increased levels of the oncogenic isoforms B and C compared to LP9 controls while levels of isoform A were relatively similar. D) LP9 mesothelial cells and HM, HP-1 and H2373 MM cells were incubated in medium containing 10% FBS (H) or medium containing 0.25% FBS (L) for 48 hours, and cell extracts were examined for FOXM1 expression by immunoblotting. Note that MM cells continue to express FOXM1 in the absence of mitogenic stimulation. E) HM cells were transfected with control siRNA (ct) and siRNA directed toward all FOXM1 splice variants (si), and 24 hr later cell extracts were examined for expression of FOXM1 by immunoblotting. Specific knockdown of FOXM1 mRNA resulted in the loss of all immunoreactive FOXM1 bands, validating the antibodies used in this study.

To define an experimental model, we then investigated the expression of FOXM1 in several human MM cell lines, using the hTERT immortalized human mesothelial cell line LP9 as a control. While HM, H2373 and several other human MM cell lines form tumors when injected into the peritoneal cavity of Fox Chase SCID mice, LP9 does not produce tumors in this background (B. Mossman and A. Shukla, unpublished data). When assessed by immunoblotting, the protein isoforms of FOXM1 differed between LP9 and MM cell lines (Fig. 2A), with LP9 expressing a species with reduced electrophoretic mobility that may represent splice isoform FOXM1A. MM cells lines express modestly higher levels of FOXM1 mRNA (Fig. 2B), and qRT-PCR for specific FOXM1 mRNA splice variants A, B and C showed that HM cells express the oncogenic FOXM1B and FOXM1C isoforms relative to splice isoform A at higher levels than LP9 cells (Fig. 2C), a result confirmed for the HP-1 and H2373 MM cell lines (data not shown). Incubation of cells in low serum conditions resulted in loss of FOXM1 expression in LP9 cells, but not in three MM cell lines (Fig. 2D), indicating that expression of FOXM1 in LP9 cells requires mitogens, but in MM cells is mitogen-independent.

FOXM1 protein isoforms are predicted to range between 75–85 kD, but isoforms that migrate with apparent molecular weights as large as 120 kD were detected by immunoblotting (Fig. 2A, D), as in other studies [32], [33]. To ensure that the antibodies used in these studies detect FOXM1, small interfering RNA (siRNA) was used to selectively extinguish FOXM1 expression, resulting in loss of all immunoreactive bands detected by the FOXM1 antibody (Fig. 2E). Due to the complexity of post-translational modifications to FOXM1 [32], [33], the precise relationship between the protein isoforms observed by gel electrophoresis and the various mRNA species is not known.

### TS Inhibits the Expression of FOXM1 in MM Cells by a Redox-dependent Mechanism

**Figure 3:**
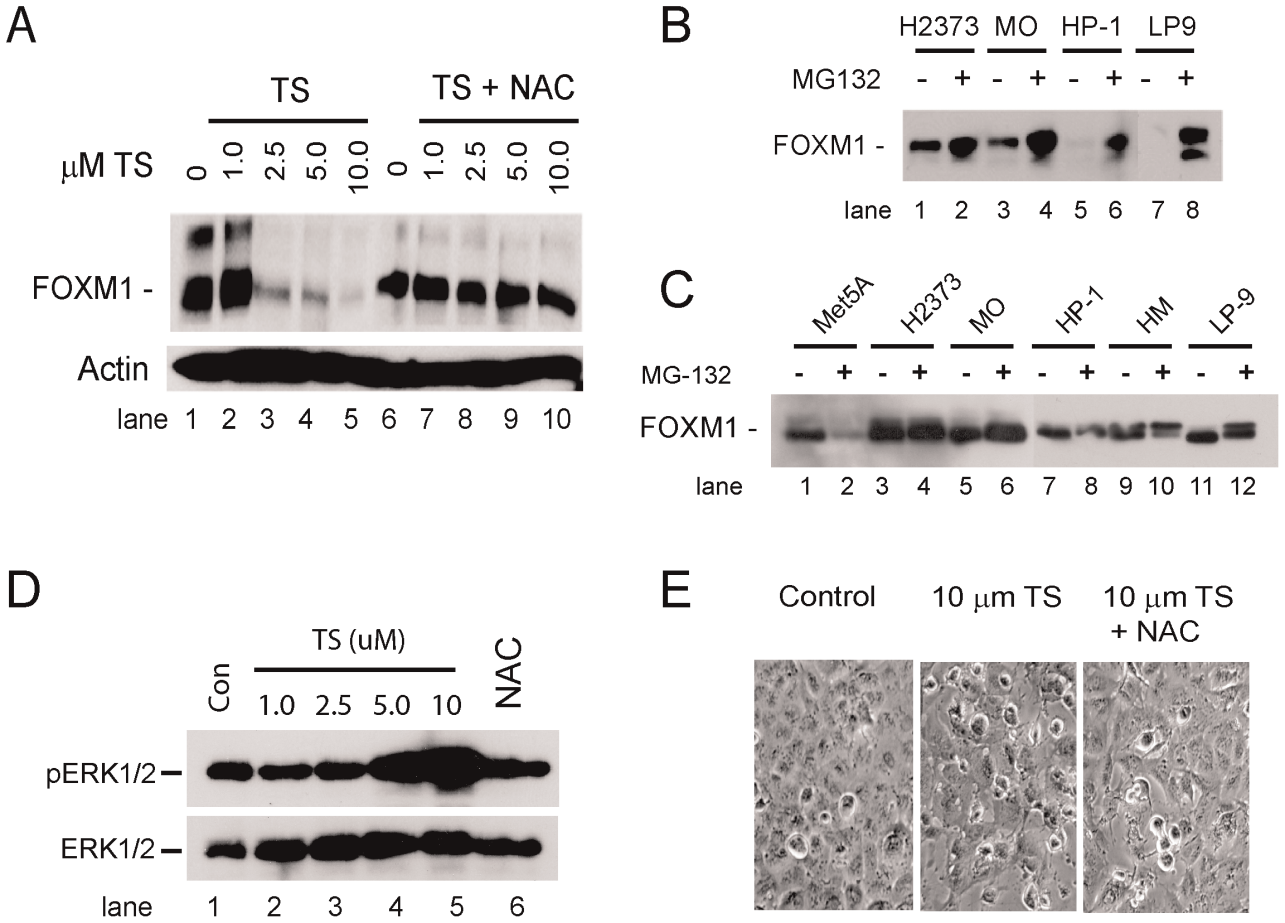
TS inhibits FOXM1 expression through a redox-dependent mechanism. A) HM cells were treated with the indicated concentration of TS for 18 hr (lanes 1–5), or were pre-incubated with 1 mM NAC for 16 hr prior to exposure to TS (lanes 6–10). Cell extracts were examined for FOXM1 expression by immunoblotting as before. B) The indicated cell lines were incubated with 2.5 µM TS, or 2.5 µM TS and 1 µM of the proteosome inhibitor MG132, for 18 hr and FOXM1 expression was examined by immunoblotting of cell extracts. C) The indicated cell lines were incubated for 16 hr with or without 10 µM MG132 as indicated and cell lysates were examined for FOXM1 expression by immunoblotting. D) HM cells were incubated with the indicated concentration of TS for 18 hr and levels of phospho-ERK1/2 (pERK1/2) and total ERK1/2 were assessed by immunoblotting. To test sensitivity of induction of phospho-ERK1/2 by TS to NAC, cells were pre-incubated with 1 mM NAC overnight and then exposed to 5.0 µM TS as before (lane 6). HM cells express significantly higher levels of ERK2 versus ERK1. E) HM cells were treated with 10 µM TS, with or without pre-incubation with 1 mM NAC, for 8 hr and examined by phase-contrast microscopy. Cell rounding, membrane retraction and other early morphological changes induced by TS were attenuated by pre-incubating HM cells with NAC.

The dose-dependent effects of TS on FOXM1 mRNA and protein expression were examined in LP9 and MM cells, and as reported by others [31], TS inhibited expression of FOXM1 by down-regulating levels of FOXM1 mRNA (not shown) and protein (Fig. 3A, lanes 2–5). However, we observed cell-type specific effects of TS in human MM cells. TS has been reported to act as a proteosome inhibitor [24], [26], but in MM and LP9 cells the proteosome inhibitor MG132 stabilized FOXM1 in the presence of TS (Fig. 3B, lanes 1–8). Treatment of LP9 cells and a panel of mesothelioma cell lines (e.g. H2373, MO, HP-1 and HM) with 10 µM MG-132 overnight did not extinguish FOXM1 expression (Fig. 3C, lanes 3–12), whereas MG-132 inhibited expression of FOXM1 in Met5A cells, a mesothelial cell line transformed by SV40 (Fig. 3C, lanes 1–2). Additionally, in contrast to the results reported for breast cancer cells [31], examination of mitogen-activated protein kinase (MAPK) signaling pathways showed TS markedly increased phospho-ERK1/2 levels in a dose-dependent fashion in HM cells (Fig. 3D). Given the sensitivity of ERK1/2 to ROS [34], we examined the effect of pre-incubating cells with N-acetyl-L-cysteine (NAC), which increases cellular glutathione levels [35], on the activity of TS. HM cells were treated overnight with 1 mM NAC, the medium was removed, and cells then were exposed to TS for 16 hr in standard growth medium as before. Expression of FOXM1, cell morphology and the phosphorylation state of the MAPK p38, JNK and ERK1/2 were examined as endpoints. Pre-incubation of cells with NAC completely blocked the effects of TS on expression of FOXM1 (Fig. 3A, lanes 6–10), activation of ERK1/2 (Fig. 3D, lane 6), and cell morphology (Fig. 3E), indicating that inhibition of FOXM1 expression by TS in MM cells is redox-dependent. TS had no effect on the phosphorylation state of JNK or p38 MAPK kinase (not shown). While prolonged activation of ERK1/2 by ROS or asbestos is incompatible with cell cycle progression and cell viability [36], blocking ERK1/2 signaling with U0126 did not rescue cells from TS-induced cytotoxicity nor did it restore FOXM1 expression (data not shown). These results indicated activation of ERK1/2 by TS is redox-dependent, but activation of the ERK signaling pathway is not an obligate step in the inhibition of FOXM1 expression in MM cells.

### Production of Mitochondrial Oxidants in MM Cells

**Figure 4:**
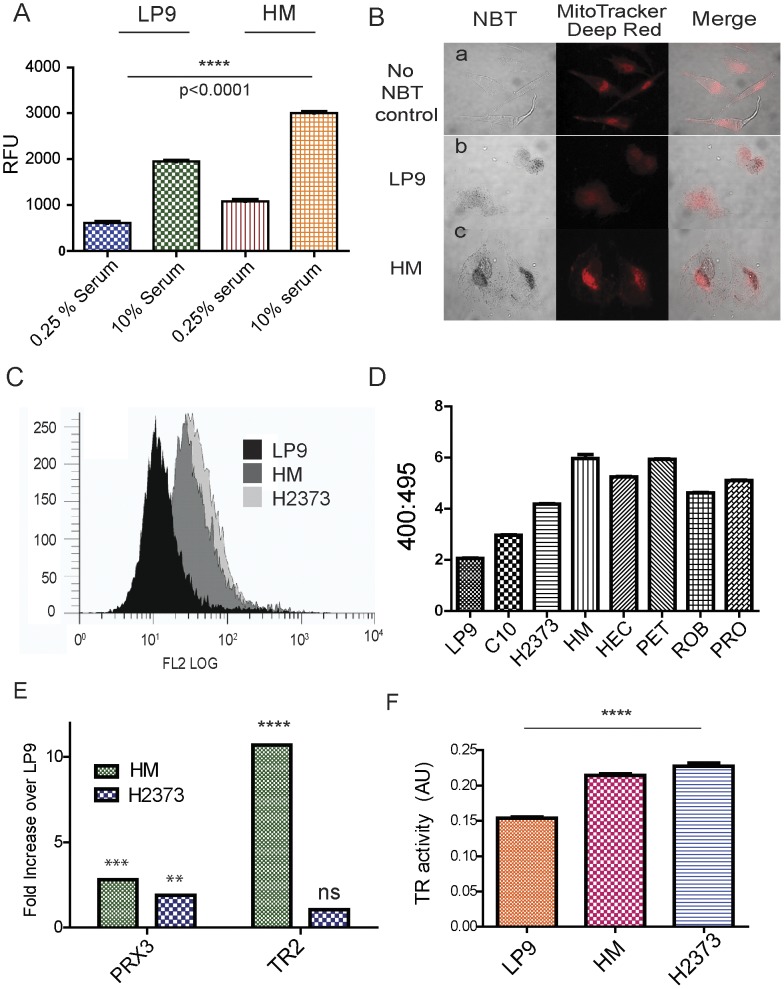
Enhanced mitochondrial superoxide production by MM cells. A) Equivalent numbers of LP9 and HM cells were deprived of serum for 72 hr, then incubated in medium with either low serum (0.25% FBS) or high serum (10% FBS) and loaded with the fluorescent reporter hydro-Cy3. After 30 min relative fluorescence was measured in triplicate; data are expressed as relative fluorescence units (RFU) +/− the standard error of the mean. B) LP9 mesothelial cells (row b) and HM MM cells (row c) were incubated with nitroblue tetrazolium (NBT) and MitoTracker Deep Red and imaged by confocal microscopy. Colocalization of the NBT and MitoTracker Deep Red signals indicated that the majority of superoxide in LP9 and HM cells is derived from mitochondria. Row a represents images of LP9 cells without staining with NBT. C) H2373 and HM MM cells and LP9 controls were loaded with MitoSOX Red for 30 min and analyzed by flow cytometry for mitochondrial superoxide production. D) The indicated cell lines were transfected with an expression vector for mitochondrial roGFP, a genetically-encoded reporter that is responsive to mitochondrial redox status. The relative redox status of the different cell lines was examined by ratiometric live cell imaging as described previously [67], [68] Higher 400∶495 ratios are indicative of an increased oxidation state in cellular mitochondria. E) Total RNA was prepared from LP9, HM and H2373 cells and relative levels of expression of PRX3 and TR2 mRNA in comparison to HPRT were determined by qRT-PCR. Data are plotted as fold-increase in the MM cell lines as compared to LP9. F) Cell extracts were prepared from LP9, HM and H2373 cells and assayed for total cellular thioredoxin reductase (TR) activity. Data are expressed as arbitrary units (AU) per 25 µg cell extract.

FOXM1 expression responds to the redox status of cells, as low levels of hydrogen peroxide increase FOXM1 expression while the antioxidant TEMPOL inhibits it [17]. Based on these observations, and the sensitivity of the effects of TS to NAC, we examined cellular oxidant production and anti-oxidant defenses as potential targets of TS. Studies with hydro-Cy3, a fluorescent probe for superoxide [37], showed under standard growth conditions HM cells produce more cellular superoxide than LP9 cells, both in low serum conditions and when stimulated with serum (Fig. 4A). Co-staining with nitroblue tetrazolium (NBT), which is reduced by superoxide and detects superoxide produced by NADPH oxidases [38], and MitoTracker Deep Red indicated that the majority of cellular superoxide was derived from mitochondria (Fig. 4B).

MitoSOX Red and flow cytometry was used to compare mitochondrial oxidant levels in MM cells to LP9 cells. MitoSOX Red, a fluorescent probe targeted to mitochondria that is responsive to superoxide and other oxidants, is considered a suitable probe for general mitochondrial oxidative stress [39]. LP9, HM and H2373 cells in log phase growth were loaded with MitoSOX Red for 30 min, trypsinized and immediately analyzed by flow cytometry, which showed MM cells produce significantly more mitochondrial oxidants than do LP9 cells (Fig. 4C). Co-staining with a DNA dye showed that increased levels of mitochondrial oxidant production occur at all phases of the MM cell cycle (data not shown). Generally all MM cell lines examined to date appear to constitutively produce 2–3 times more mitochondrial oxidants than do LP9 cells (Fig. 4A and C), and ratiometric imaging with a redox-sensitive green fluorescent protein (roGFP) targeted to mitochondria confirmed this general phenotypic property in a representative panel of MM cell lines (Fig. 4D).

When compared to LP9 cells, MM cells appear to have adapted to enhanced levels of mitochondrial oxidant production by up-regulating expression of thioredoxin reductase 2 (TR2) and peroxiredoxin 3 (PRX3), components of a major mitochondrial anti-oxidant network (Fig. 4E). PRX3 is responsible for 90% of the peroxidase activity in mitochondria [28], [29], and both TR2 and PRX3 are known to be over-expressed in human MM tumors [40], [41], [42]. Total cellular thioredoxin reductase (TR) activity also was significantly increased in MM cells as compared to LP9 cells (Fig. 4F). Together these results indicated that the mitochondria of MM cells consistently produce significantly higher levels of mitochondrial oxidants, and that MM cells adapt to this pro-oxidant state by up-regulating the TR2/TRX2/PRX3 antioxidant network.

### PRX3 is a Redox-dependent Target of TS

**Figure 5:**
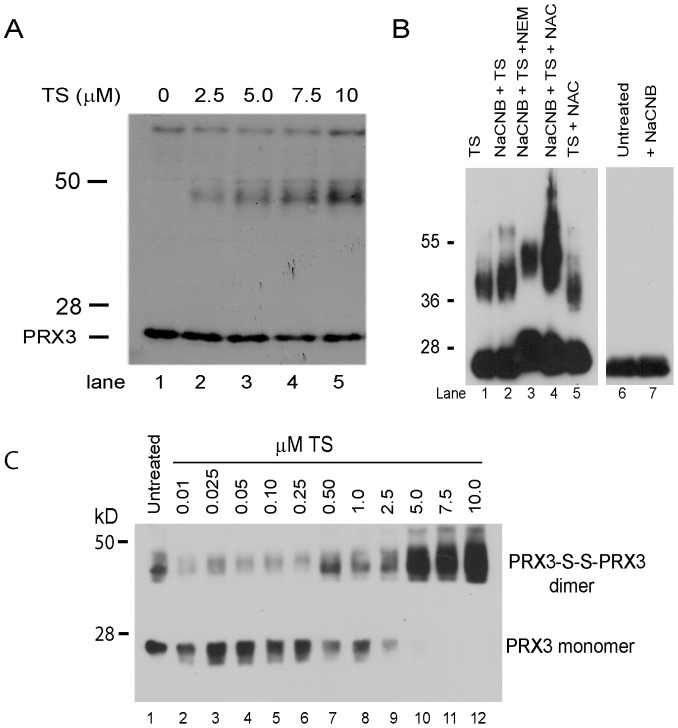
The electrophoretic mobility of PRX3 is modified by TS. A) HM cells were treated with the indicated concentration of TS for 18 hr, and cell extracts were prepared, heated in SDS sample buffer at 95°C for 10 min, resolved by gel electrophoresis, and PRX3 expression was assessed by immunoblotting. Treatment of HM cells with increasing doses of TS caused the formation of a modified species of PRX3 that migrated with an apparent molecular weight of about 45–50 kD. B) Recombinant human PRX3 (rPRX3) was incubated with 10 µM TS in solution in vitro using conditions described by Chiu and colleagues [43], and samples were resolved by gel electrophoresis and examined by immunoblotting. Pre-treatment of rPRX3 with sodium cyanoborohydride (NaCNB) accentuated the reactivity of TS (lane 2) whereas pre-incubation with NAC inhibited it (lane 5). Addition of TS and N-ethyl-maleimide (NEM) or NAC to rPRX3 treated with NaCNB resulted in multiple species of immunoreactive PRX3 (lanes 3 and 4), whereas treatment with NACNB alone (lane 7) had no effect. C) HM cells were treated with the indicated concentration of TS for 18 hr, and cell extracts were prepared in the absence of reducing agents as described previously [69]. Samples (20 µg protein/lane) were resolved under denaturing but not reducing conditions, and the relative distribution of PRX3 monomers to dimers was assessed by immunoblotting.

When examining the dose-dependent effects of TS on the cellular TR network, we observed that treatment of MM or LP9 cells with TS at concentrations at or above 2.5 µM altered the electrophoretic mobility of PRX3, producing modified forms of PRX3 with apparent molecular weights ranging from 44–50 kD (Fig. 5A), approximately the molecular weight of disulfide-bonded PRX3 dimers. However, these immunoreactive species were maintained when heated to 95°C for more than 10 min in SDS sample buffer with dithiothreitol (DTT), indicating the modified species of PRX3 are resistant to denaturation by detergents or reduction by DTT. Based on experiments with the bacterial protein TipAS, in which cysteine residues are directly adducted by reactive dehydroalanine moieties in TS [43], recombinant human PRX3 (rPRX3) was incubated with TS in vitro. These experiments showed TS induces a modified form of rPRX3 in vitro with similar electrophoretic mobility as was observed for PRX3 in cell extracts (Fig. 5B). Reduction of rPRX3 with sodium cyanoborohydride increased modification of rPRX3 by TS (Fig. 5B, lane 2), while NAC inhibited modification of PRX3 by TS (Fig. 5B, lane 5), supporting the possibility that TS directly adducts cysteine residues in PRX3. It does not appear that TS quantitatively adducts all cysteine residues in PRX3, for the thiol-reactive agent N-ethyl-maleimide (NEM) altered the mobility of the TS:rPRX3 complex (Fig. 5B, lane 3). The dehydroalanine residues in TS have been shown to bind 3–4 free cysteines [43], and addition of NAC along with TS to PRX3 reduced by sodium cyanoborohydride resulted in multiple species of PRX3 with altered mobility. Under no condition was rPRX3 quantitatively modified by TS in vitro.

Like other 2-Cys peroxiredoxins, PRX3 functions as an obligate homodimer, and disulfide-bonded dimers are generated during the resolving step of the catalytic cycle [28], [44]. Using denaturing but not reducing gel electrophoresis conditions that preserve disulfide bonds, the relative distribution of PRX3 monomers was compared to PRX3-S-S-PRX3 dimers, which can only be generated during the PRX3 catalytic cycle. In the presence of increasing concentrations of TS, monomer PRX3 was detected until concentrations of TS at or above 2.5 µM (Fig. 5C), when all PRX3 was observed to migrate at the apparent molecular weight of PRX3 dimers. These results indicate complete loss of reduced PRX3 monomers, either through direct modification by TS or disulfide bond formation between homodimeric subunits during the PRX3 catalytic cycle, and are indicative of induction of severe mitochondrial oxidative stress.

### Gentian Violet Potentiates the Formation of Modified PRX3

**Figure 6:**
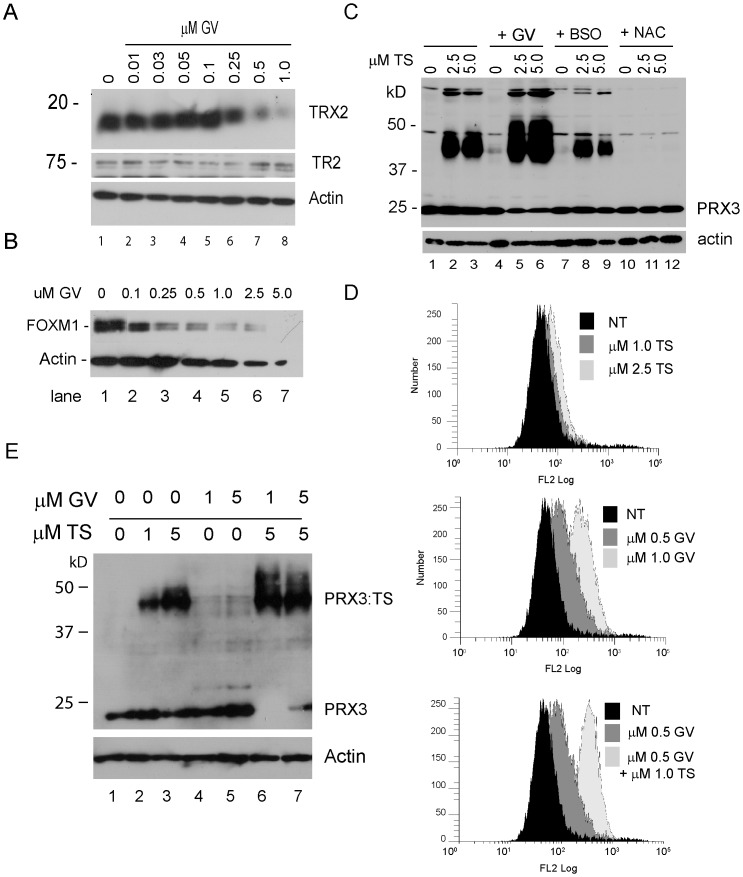
GV accentuates modification of PRX3 by TS. A) HM cells were treated with the indicated concentration of GV for 18 hr, cell extracts were prepared, and expression of TRX2 and TR2 was assessed by immunoblotting. Actin was used as a loading control. B) HM cells were treated with the indicated concentration of GV for 16 hr, cell extracts were prepared and examined for FOXM1 expression by immunoblotting. Actin was used as a loading control. C) HM cells were treated with the indicated concentration of TS, TS plus GV, or TS after pre-incubation with either BSO or NAC. Cell extracts were prepared and assessed for PRX3 expression by immunoblotting as before. Actin was used as a loading control. D) HM cells were treated with the indicated concentration of TS, GV or TS plus GV for 18 hr, loaded with MitoSOX Red for 40 min, and examined by flow cytometry. Data are expressed as the log of the relative fluorescent signal (FL2) versus the number of cells. NT indicates no treatment. E) HM cells were treated with TS, GV or TS plus GV at the indicated concentrations, and modification of PRX3 was examined by immunoblotting as before. Note that under these conditions the electrophoretic mobility of the entire cellular pool of PRX3 was modified in cells treated with TS plus GV.

The cationic triphenylmethane gentian violet (GV) has antitumor activity that is redox-dependent [30], [45]. Recently GV was shown to impair mitochondrial antioxidant capacity by inhibiting the expression of TRX2, but not TR2 [30]. In mitochondria TRX2 is the oxidoreductase that reduces disulfide-bonded PRX3 dimers, and thereby is required for regenerating reduced PRX3 during the 2-Cys peroxiredoxin catalytic cycle [46]. In HM cells, GV inhibited the expression of TRX2 in a dose-dependent manner, with significant inhibition at doses as low as 0.5 µM (Fig. 6A). GV had no effect on the expression of TR2, but like TS, GV inhibited the expression of FOXM1 (Fig. 6B).

When combined with TS, GV resulted in marked increases in the levels of modified PRX3 (Fig. 6C, lanes 5–6). When HM or other MM cells were pre-incubated with NAC, no modified form of PRX3 was observed (Fig. 6C, lanes 11–12, and data not shown). L-buthionine-*S*,*R*-sulfoximine (BSO), an inhibitor of cellular glutathione synthesis [35], had no significant effect on modification of PRX3 by TS (lanes 8–9). Analysis of mitochondrial redox status using the redox-responsive fluorescent dye MitoSOX Red and flow cytometry showed TS induces increased levels of mitochondrial oxidants at 2.5 µM or above (Fig. 6D), in excellent agreement with the concentrations of TS that promote modification of PRX3. At concentrations that inhibit expression of TRX2 or above (e.g. >0.5 µM), GV increased mitochondrial oxidant levels, and when added together, TS and GV caused production of even higher levels of mitochondrial oxidants (Fig. 6D). GV alone did not alter the electrophoretic mobility of PRX3 (Fig. 6E, lanes 4–5), but at markedly cytotoxic doses of GV and TS the electrophoretic mobility of the entire cellular pool of PRX3 was modified (Fig. 6E, lanes 6–7). Under these conditions all immunoreactive species of PRX3 migrated in excess of 44–50 kD under denaturing and reducing conditions.

**Figure 7:**
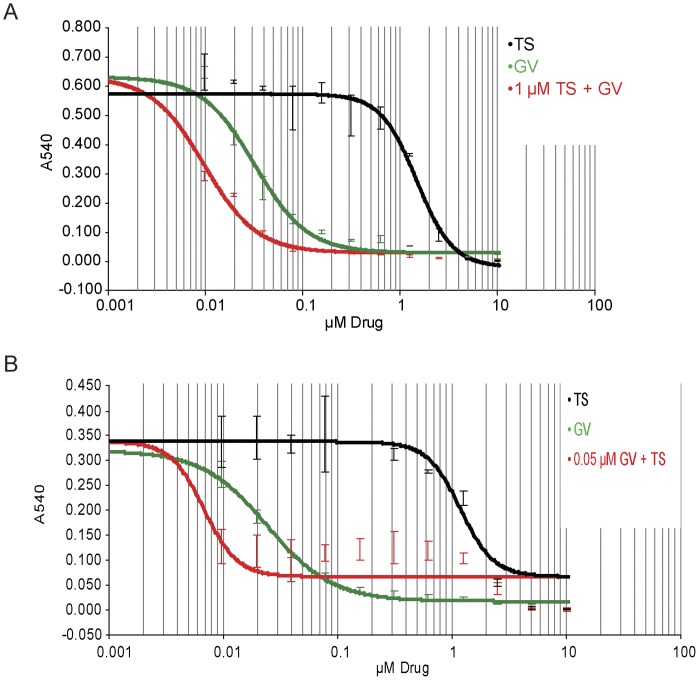
GV potentiates the cytotoxicity of TS in MM cells. A) HM cells were plated in duplicate in 96 well plates, and treated with either increasing concentrations of GV or TS alone, or 1.0 µM TS with increasing concentrations of GV. After 4 days cells were stained with crystal violet and total cellular mass was assessed by absorbance of methanol-soluble dye at 540 nM. Using the four parameter non-linear regression model in Gen5 software (BioTek Instruments), 1 µM TS with increasing [GV] was estimated to be approximately 3.3-fold more potent than increasing concentrations of GV alone. B) HM cells were plated in 96 well dishes and treated with the indicated concentrations of TS and GV, or with 0.05 µM GV with increasing concentrations of TS. After 4 days total cell mass was quantified by staining with crystal violet as above. A constant concentration of 0.05 µM GV with increasing [TS] was estimated to be approximately 175 times more potent than increasing concentrations of TS alone.

Based on the ability of GV to enhance modification of PRX3, we tested the effect of GV on cell viability in response to exposure to TS. HM cells were plated in 96 well dishes and exposed to 1.0 µM TS alone, approximately half the ID_50_ (2.3+/−0.3 µM), or 1.0 µM TS and increasing concentrations of GV. Using a four parameter linear regression model, the combination of 1 µM TS plus GV was 3.3-fold more potent than GV alone (Fig. 7A), and in the reverse format 0.05 µM GV plus TS was 175-fold more potent than TS alone (Fig. 7B). Phenylethyl isothiocyanate (PEITC), a redox-active agent which affects PRX3 oxidation state [47], also enhanced the cytotoxic activity of TS (not shown). We conclude from these studies that TS disables the TR2-TRX2-PRX3 network by covalently adducting PRX3, and that GV enhances adduction of PRX3 by TS by inhibiting expression of TRX2. The mechanism by which disruption of PRX3 activity and increased levels of mitochondrial oxidants inhibit FOXM1 expression is not known.

## Discussion

The role of ROS in cell physiology is complex, with the source, species, rate of production, steady state concentration and subcellular location contributing to its effects on specific targets that control cell fate decisions [14]. As the primary source of cellular ROS, mitochondria play a central role in redox signaling [48]. Mitochondria from tumor cells commonly display altered morphology, changes in membrane potential, and perturbations in energy metabolism, consequences of neoplastic transformation and uncontrolled cell proliferation [48], [49], [50]. Enhanced consumption of glucose during aerobic glycolysis is associated with increased mitochondrial superoxide production [51]. While it is possible that superoxide may signal directly, it more likely is rapidly dismutated by MnSOD to H_2_O_2_, which is capable of diffusing to the cytoplasm and modulating signaling cascades that regulate mitogenesis, cell migration, drug resistance and other processes of malignancy (reviewed in [52]). There are a large number of studies that have targeted perturbations in ROS metabolism as a therapeutic strategy in cancer, and induction of intolerable levels of oxidative stress generally appears to be a more effective strategy than blocking oxidant production [53], [54].

Based on studies that show FOXM1 responds to cellular redox status, albeit through unknown mechanisms [17], we explored the effect of cellular redox status on the activity TS in a cell culture model of human MM. MM shares several features with “reactive oxygen-driven tumors” [15], and the studies here show MM cells in culture constitutively produce 2–3-fold more mitochondrial oxidants than non-transformed LP9 mesothelial cells. We expect that responses to TS will differ between tumor cell types based on specific perturbations in oxidant metabolism and the array of redox-responsive targets expressed in different cell types. For example, in breast cancer cells TS inhibits activation of ERK1/2 [31], whereas in MM cells TS activates ERK1/2, and activation is sensitive to the antioxidant NAC (Fig. 3C). MM cells apparently adapt to a pro-oxidant mitochondrial state by up-regulating expression of TR2 and PRX3, both of which are over-expressed in human mesothelioma [40], [41], [42].

Our results indicate TS disables the TR2/TRX2/PRX3 mitochondrial anti-oxidant pathway by covalently adducting cysteine residues in PRX3, similar to the adduction of a cysteine residue by TS in the bacterial protein TipAS [43]. TS covalently binds TipAS with a 1∶1 stoichiometry, and the peptide harboring the modified cysteine peptide is increased in MW by 1664 Da, in excellent agreement with the MW of TS [43]. The covalent modification of a cysteine residue in TipAS by TS is mediated by dehydroalanine moieties and is resistant to reducing and denaturing agents [43], as is the modified form of human PRX3 induced by TS. As for TipAS, PRX3 modification by TS is sensitive to competition by free thiols. TS has three dehydroalanine residues capable of reacting with thiols [43], and PRX3 dimers have six cysteine residues (three per monomer), and one plausible interpretation of our results is that TS covalently cross links PRX3 homodimers through cysteine residues (or some other amino acid). The peroxidatic cysteine residues in peroxiredoxins are among the most reactive protein thiols in cells [28], [29], [41], [44], [47], [52], suggesting the cysteine residues intimately involved in peroxidase activity may be preferred targets.

Inhibition of TRX2 expression by GV markedly enhanced modification of PRX3 by TS (Fig. 6). Since TRX2 is the oxidoreductase required for reduction of PRX3 disulfide-bonded dimers, and only a small fraction of rPRX3 was modified by TS in vitro, this suggests that catalytic intermediates with specific oxidation states that accumulate in the PRX3 reaction cycle in the absence of TRX2 may be preferred substrates for TS. Each head-to-tail PRX3 dimer has two reaction centers composed of the peroxidatic cysteine in one subunit in apposition to the resolving cysteine in the other [28], [29]; these two catalytic centers are oriented in opposite directions in the PRX3 dimer and may function independently. In PRX3 dimers containing one disulfide bond, TS may interact with thiols in the other catalytic site, or with Cys65, which has no known role in catalysis. Since we did not observe a species of PRX3 with an increase in apparent molecular weight of about 2 kD, we expect that the complex structure of TS promotes specific binding to surfaces of the PRX3 dimer, thereby orienting two or more dehydroalanine moieties in TS in close proximity to reactive thiols in each subunit, leading to cross-linking and enzymatic inactivation of PRX3.

In higher concentrations of TS, PRX3 also becomes hyperoxidized (not shown), but it is not known if this intermediate is a preferential target of TS. Indeed, the modified forms of PRX3 do not migrate as a single species, and mass spectrometry and mutagenesis of PRX3 will be required to identify the amino acid residues adducted by TS in vitro, in cells, and in response to treatment with GV. Moreover, we expect that TS reacts with other redox-dependent targets in MM cells, and global analysis of protein modification by TS will be required to identify additional proteins that may play a role in the anti-tumor activity of TS. Our present results indicate, however, than PRX3 is more reactive with TS than either PRX1 or PRX2 (not shown), although preferential accumulation of TS in mitochondria might account for differences in sensitivity.

PRX3 is consistently up-regulated in prostate cancers [55], is over-expressed in greater than 90% of hepatocellular carcinomas [56], and increases in abundance during malignant progression of cervical cancer [57]. Inhibition of PRX3 expression in breast cancer cells induces cell cycle arrest and impairs cell proliferation [58]. Since elevated expression of PRX3 is linked to resistance to apoptosis, inhibiting the peroxidase activity of PRX3 has been proposed as a therapeutic target in cancer [29]. Here we have detected a link between mitochondrial oxidant metabolism and FOXM1 expression, and our present evidence indicates that TS acts on this axis through covalent adduction of PRX3, leading to increases in mitochondrial oxidant production and/or general mitochondrial oxidative stress. Of particular interest is the mechanism by which high levels of oxidants impair FOXM1 expression, and one intriguing possibility is that this phenotypic response results from redox-dependent activation of the anti-proliferative transcription factor FOXO3a, which is required to elicit responses to many chemotherapeutic agents [5], [59], [60]. Since FOXO3a and FOXM1 regulate many of the same genes, it is possible that a redox-dependent interplay between the activation of these transcription factors regulates responses to TS. Suppression of FOXM1 expression does not appear to be linked to activation of ERK1/2 by TS, for inhibition of ERK activity with U0126 in the presence of by TS did not restore FOXM1 expression (not shown).

**Figure 8:**
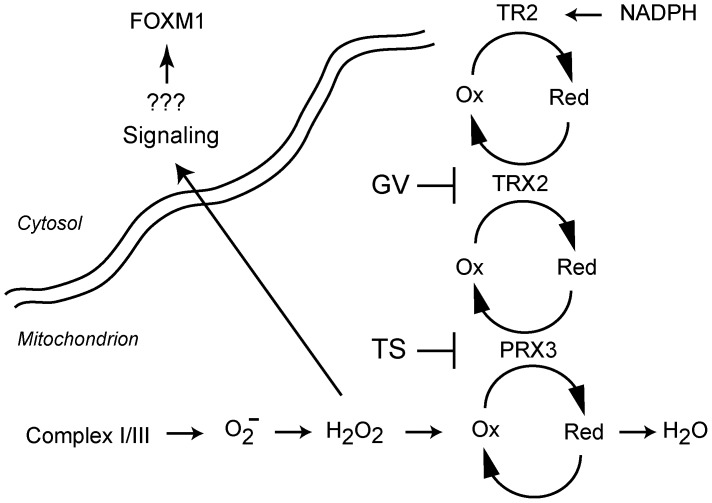
Cooperative effects of GV and TS on the TR2/TRX2/PRX3 antioxidant network. Superoxide from the electron transport and chain and other sources is converted by MnSOD to H_2_O_2_, which is then metabolized by PRX3 to water. Disruption of PRX3 activity by covalent adduction of cysteine residues by TS increases mitochondrial oxidant levels, which repress FOXM1 expression through unknown mechanisms. Inhibition of TRX2 expression by GV potentiates the activity of TS, increasing its cytotoxicity. Combinatorial approaches to inactivating the TR2/TRX2/PRX3 antioxidant network provide an approach for disabling an important adaptive response in MM, and may enhance the sensitivity of MM to conventional chemotherapeutic drugs.

There are several important implications to these findings. A number of approaches have emerged for exploiting FOXM1 as a therapeutic target in other malignancies [61], [62], [63], [64]. FOXM1 is an attractive target since it is not expressed in quiescent cells [4], and is required for cell cycle progression and viability in many tumor cell types. TS alone has modest anti-tumor activity [21], and the observation that low doses of GV markedly potentiate modification of PRX3 by TS provides a combinatorial approach for inactivating the mitochondrial TR2/TRX2/PRX3 anti-oxidant network and inducing cytotoxicity (Fig. 8). Disabling this antioxidant network also may increase sensitivity to conventional drugs that induce apoptosis. Further work will be required to optimize the ratio of TS to GV that provides selectivity for tumor versus normal cells, and to test these approaches in models that more closely approximate tumor environments in vivo, which no doubt differ significantly from cell culture conditions.

Our approach may be particularly appropriate for intractable tumor types characterized by high levels of mitochondrial oxidant production. For example, melanoma cell lines have been grouped into two categories based on the expression of anti-apoptotic and anti-oxidant genes, including *PRDX3* [65]. TS induces apoptosis in melanoma cells in a redox-dependent manner, for NAC rescues melanoma cells from TS toxicity [27], but the role of PRX3 in cell death in this context is not known. We suggest documenting the expression levels of PRX3 may prove useful in identifying malignancies susceptible to combinations of TS and GV. Finally, GV is safe in humans [66], and TS has no overt toxicity in animal models [21], suggesting that once optimal ratios of the two compounds that exploit the differences in mitochondrial oxidant metabolism between normal mesothelial cells and MM tumor cells are refined, translation to clinical applications might proceed rapidly.

## Materials and Methods

### Cell Lines and Cell Culture

Four pleural human MM cell lines were used in these studies. HMESO1 (HM) was obtained from J. Testa (Fox Chase Cancer Center, Philadelphia, PA); this cell line was originally isolated by Reale and colleagues [70]. HP-1 and H2373 were established from human MMs after surgical resection [71], and were verified to be mesothelial using a calretinin antibody. Morphologically, HM and HP-1 are epithelioid, while H2373 is fibrosarcomatoid. LP9, a human mesothelial cell line immortalized with hTERT, was obtained from J. Rheinwald (Dana Farber Cancer Institute, Boston, MA). Cell lines were validated by STR DNA fingerprinting using the Promega CELL ID System (Promega, Madison, WI). The STR profiles are of human origin, and did not match known DNA fingerprints in the Cell Line Integrated Molecular Authentication database (http://bioinformatics.istge.it/clima/), but will serve as a reference for future work. In several experiments, the MM cell lines HEC, PET, ROB and PRO established by C. Verschraegen (Vermont Cancer Center) were also examined. Cells were maintained in DMEM-F12 with hydrocortisone, insulin, transferrin, and selenite with 10% fetal bovine serum (FBS, GIBCO) as previously described [72]. For low serum conditions, cells were incubated for 48–72 hours in complete medium containing 0.25% FBS.

### Immunoblotting

Cell lysates were prepared as previously described [34]. Protein concentrations were determined using Bradford assays (Bio-Rad, Hercules, CA). Lysates (20–25 µg protein/well) were resolved by SDS-PAGE and prepared for immunoblotting as previously described [69]. Blots were incubated at 4°C overnight with rabbit anti-FOXM1 K19 polyclonal antibody (Santa Cruz Biotechnology, Santa Cruz, CA) at a 1∶500 dilution in blocking buffer, and after washing protein bands were visualized with the Western Lightning chemiluminescent detection system (Perkin Elmer, Waltham, MA) using secondary antibodies coupled to horse radish peroxidase. Blots were re-probed with a mouse anti-actin antibody (Millipore, Billerica, MA) to verify equal protein loading.

### FOXM1 Isoform-specific PCR

The detection of specific FOXM1A, B and C mRNA splice variants via RT-PCR was performed as previously described [73].

### FOXM1 siRNA-mediated Knockdown

Cells were grown to 70% confluence, and medium was changed to serum-free OPTI-MEM before preparing siRNA stocks. Mixtures of FOXM1 siRNA (GGACCACUUUCCCUACUUU) [17] and scramble control (GCUCCUUUCGUCUCACAUAUU) were prepared in OPTI-MEM as described by the manufacturer (Dharmacon, Lafayette, CO). Cells were transfected with siRNA using Lipofectamine 2000 (Invitrogen, Carlsbad, CA) in OPTI-MEM; after 4–6 hours, FBS was added to a final concentration of 10%. Media was changed to DMEM-F12 complete media 24 hrs after initial transfection. Cell extracts were prepared for immunoblotting as before [34].

### Measurement of ROS Production

Hydro-Cy3 [37], a fluorescent probe specific for intracellular superoxide and hydroxyl radicals, was a gift from N. Murthy (Georgia Institute of Technology, Atlanta, GA). Cells were plated in black clear-bottom 96-well microplates (Corning, Lowell, MA), incubated in starvation medium for 48 hrs prior to stimulation with complete medium containing 0.25% or 10% FBS for 45 minutes at 37°C. Cells were then washed in phenol red- and serum-free DMEM-F12 medium, loaded with 5 µM hydro-Cy3 prepared in phenol red- and serum-free DMEM-F12, and read at 535_ex_/560_em_ every minute for 30 minutes using a BioTek Synergy H4 Hybrid microplate reader (BioTek Instruments, Winooski, VT).

### Thioredoxin Reductase Activity Assay

Cells were scraped from culture dishes in 50 mM Tris/Cl, pH 7.5, with 1 mM EDTA, sonicated, and centrifuged at 14,000 rpm for 10 min. Assays contained 20 µg lysate protein, 50 mM Tris/Cl, pH 7.5, 1 mM EDTA, 8 mM NADPH, 6 mg/ml insulin with or without purified recombinant thioredoxin. Reaction mixtures (80 µl) were incubated at 37°C, and after 1 hr reactions were terminated by adding 920 µl of 8 M guanidine-HCl containing 1 mM DTNB and read at 412 nm. Activity is expressed as A_412_ per µg protein after subtraction of reaction controls without substrate.

### RNA Preparation and Gene Expression

Trizol total RNA extraction and subsequent DNase treatment were performed as described by the manufacturer (Qiagen, Valencia, CA). Patient tumor tissues were homogenized using a Ultraturex homogenizer. cDNA was prepared using the High Capacity cDNA Reverse Transcription kit (Applied Biosystems, Foster City, CA). Assays for RNA expression were done in triplicate using Assay On Demand (Applied Biosystems) for FOXM1 (Hs01073586_m1) and HPRT (Hs02800695_m1). Changes in relative mRNA expression levels in MM cell lines were compared to those in LP9 cells, and in tumor specimens to matched normal mesothelium. qPCR was performed on an Applied Biosystems (ABI) Prism 7900HT Sequence Detection System using the SDS v2.2 software.

### Patient Materials and Immunostaining of Tissue Arrays

Tissue microarrays were obtained through the Mesothelioma Virtual Tissue Bank; they included duplicate paraffin-embedded sections for 46 tumors, (28 epithelial/epithelioid, 9 biphasic, 3 sarcomatoid, 4 papillary and 2 desmoplastic specimens). The histological diagnosis was not specified for 13 specimens, and these were not included in our analysis. Arrays were stained by immunohistochemistry (IHC) as described previously [64], using polyclonal FOXM1 antibody C-20 (Santa Cruz Biotechnology) at dilutions of 1∶1000, 1∶2000 and 1∶3000. Specimens stained with the 1∶3000 dilution showed predominantly nuclear staining and were scored by two independent observers: 0 =  no positive nuclei, 1 = <5% positive nuclei, 2 = >5% and <50% positive nuclei, and 3 = >50% positive nuclei.

### Inhibitors

The cationic triphenylmethane gentian violet (GV) was a kind gift from J. Arbiser (Emory University, Atlanta, GA). Thiostrepton was from EMD Biochemicals.

### Cell Growth Assays

Cells were plated in 96-well plates at a density of 1500 cells per well. The following day, cells were treated with test compounds in complete medium with 10% FBS. After 4 days cells were washed with PBS, fixed in 3.7% para-formaldehyde (PFA) and stained for 30 min with 0.1% crystal violet in water. To quantify crystal violet staining, the dye was dissolved in 100% methanol absorbance was read at 540 nm. Signals were normalized by subtracting the background signal from wells treated in the same fashion, but with no cells. To determine the relative potency (REP) of combinations of drugs to individual compounds the data were plotted using a 4-parameter non-linear regression model with the Gen5 software, using responses to the parent compound as the reference curve (BioTek Instruments, Winooski, VT).

### MitoSOX Red Flow Cytometry

After indicated treatments cells were loaded with 5 µM MitoSOX Red in tissue culture medium for 30 minutes. Cells were washed with Hanks buffered salt solution with calcium and magnesium (HBSS), collected by brief trypsinization, centrifuged and washed twice in HBSS, and re-suspended in 1% bovine serum albumin (BSA) in HBSS and analyzed by flow cytometry. To measure oxidized MitoSOX Red, cells were excited at 488 nm and emission was collected in the FL2 channel. No dye and menadione-treated cells were used as controls for each experiment (data not shown).

### Measurement of Mitochondrial Redox Status by Mito-roGFP2

Cells were plated in 35 mm glass bottom imaging dishes (MatTek, Ashland, MA) and transiently transfected with mitochondrial targeted pEGFP-R12 (roGFP2) using Lipofectamine 2000 (Invitrogen). The following day media was changed to CO_2_- independent media (Invitrogen) supplemented with all other components of complete MM media and imaged on a Nikon Ti-E inverted microscope with a 100X 1.49 NA objective in a heated environmental chamber. To determine the oxidation state of the probe, fluorescence images were collected with an Andor iXon X3 EMCCD camera (Andor Technology, Belfast, UK) after excitation at 400 nm and 495 nm using a diode illuminator (Lumencor, Beaverton, OR); emission was collected at 525 nm for both excitation wavelengths. Individual cells were imaged every minute for 30 minutes and the ratio of emission from 400 (oxidized) and 495 (reduced) was measured to determine the amount of oxidized probe in each cell line tested. Quantification of signals was determined using the NIS-Elements software (Nikon Instruments, Melville, NY) and is graphically depicted as the mean of the 400/495 ratio.

### Confocal Microscopy

Cells were plated on glass coverslips in 35 mm tissue culture dishes in complete media. 2.0 µg/ml of nitroblue tetrazolium (Sigma) was added to each plate in HBSS for 40 min. A final concentration of 250 nM of Mitotracker Deep Red (Invitrogen) was added directly to each plate for the final 20 min of NBT staining. Cells were subsequently washed and fixed in 3.7% PFA and mounted on glass slides in mounting media. Images were collected on a Zeiss LSM 510 META confocal laser scanning imaging system using a Plan-Neofluar 40X objective. NBT images were visualized using differential interference contrast (DIC) while MitoTracker Deep Red was visualized through excitation at 633 nM and emission collection at 650 nm.

### Incubation of Recombinant PRX3 with TS

Recombinant human PRX3 (Prospec Biochemicals, East Brunswick, NJ) was treated with TS as described by Chui and colleagues [43]. Briefly, 2 µg of rPRX3 protein in 20 mM Tris-HCl at pH 8 and 10% glycerol was reduced with sodium cyanoborohydride (0.5 µl of 1 mM freshly made stock/20 µl reaction mix). After incubating for 5 min at room temperature, excess sodium cyanoborohydride was removed by adding 1 µl acetone. After the addition of TS to a final concentration of 5 µM, the reaction was incubated at room temperature for another 15 min. Selected samples were treated with NAC (final concentration of 1 mM) or alkylated with N-ethyl-maleimide for 15 minutes at room temperature. Samples were then heated in SDS sample buffer and subjected to SDS-PAGE and immunoblotting blotting as described above.

### Statistics

For *in vitro* experiments, at least two independent replicates were performed (n = 2 to 4 samples/group/experiment). Statistical significance was evaluated by analysis of variance (ANOVA) using the Student Neuman-Keul’s procedure for adjustment of multiple pairwise comparisons between treatment groups. Values of p<0.05 were considered statistically significant. One asterisk indicates a p value <0.05, two asterisks p<0.01, three asterisks p<0.001, and four asterisks p<0.0001.
